# Bayesian belief network modelling approach for predicting and ranking risk factors for malaria infections among children under 5 years in refugee settlements in Uganda

**DOI:** 10.1186/s12936-023-04735-8

**Published:** 2023-10-04

**Authors:** Henry Musoke Semakula, Song Liang, Paul Isolo Mukwaya, Frank Mugagga, Denis Nseka, Hannington Wasswa, Patrick Mwendwa, Patrick Kayima, Simon Peter Achuu, Jovia Nakato

**Affiliations:** 1 Department of Geography, Geo-informatics and Climatic Sciences, Makerere University, P.O Box 7062, Kampala, Uganda; 2 Department of Environmental and Global Health, College of Public Health and Health Professions, University of Florida, 2055 Mowry Rd, Gainesville, FL 32610 USA; 3 Department of Environmental Health Sciences, School of Public Health & Health Sciences, University of Massachusetts, Amherst, 01003 USA; 4 National Environmental Management Authority (NEMA), Plot 17/19/21 Jinja Road, P.O. Box 22255, Kampala, Uganda; 5 Department of Horticulture and Food Security, Jomo Kenyatta University of Agriculture and Technology, P.O. Box 62000-00200, Nairobi, Kenya

**Keywords:** Bayesian belief network, Children, Malaria, Ranking, Refugees, Risk factors, Settlements, Uganda

## Abstract

**Background:**

Malaria risk factors at household level are known to be complex, uncertain, stochastic, nonlinear, and multidimensional. The interplay among these factors, makes targeted interventions, and resource allocation for malaria control challenging. However, few studies have demonstrated malaria’s transmission complexity, control, and integrated modelling, with no available evidence on Uganda’s refugee settlements. Using the 2018–2019 Uganda’s Malaria Indicator Survey (UMIS) data, an alternative Bayesian belief network (BBN) modelling approach was used to analyse, predict, rank and illustrate the conceptual reasoning, and complex causal relationships among the risk factors for malaria infections among children under-five in refugee settlements of Uganda.

**Methods:**

In the UMIS, household level information was obtained using standardized questionnaires, and a total of 675 children under 5 years were tested for malaria. From the dataset, a casefile containing malaria test results, demographic, social-economic and environmental information was created. The casefile was divided into a training (80%, n = 540) and testing (20%, n = 135) datasets. The training dataset was used to develop the BBN model following well established guidelines. The testing dataset was used to evaluate model performance.

**Results:**

Model accuracy was 91.11% with an area under the receiver-operating characteristic curve of 0.95. The model’s spherical payoff was 0.91, with the logarithmic, and quadratic losses of 0.36, and 0.16 respectively, indicating a strong predictive, and classification ability of the model. The probability of refugee children testing positive, and negative for malaria was 48.1% and 51.9% respectively. The top ranked malaria risk factors based on the sensitivity analysis included: (1) age of child; (2) roof materials (i.e., thatch roofs); (3) wall materials (i.e., poles with mud and thatch walls); (4) whether children sleep under insecticide-treated nets; 5) type of toilet facility used (i.e., no toilet facility, and pit latrines with slabs); (6) walk time distance to water sources (between 0 and 10 min); (7) drinking water sources (i.e., open water sources, and piped water on premises).

**Conclusion:**

Ranking, rather than the statistical significance of the malaria risk factors, is crucial as an approach to applied research, as it helps stakeholders determine how to allocate resources for targeted malaria interventions within the constraints of limited funding in the refugee settlements.

**Supplementary Information:**

The online version contains supplementary material available at 10.1186/s12936-023-04735-8.

## Background

Malaria, a mosquito-borne disease continues to be a major public health concern in Africa with longstanding infections leading to significant morbidity, and mortality especially among children under 5 years [1]. By 2021, approximately 234 million malaria cases, and 593,000 deaths occurred in Africa [2], imposing a heavy burden on human societies, negatively impacting community welfare, and constraining socio-economic development [3]. Some malaria related deaths in Africa have also been attributed to the COVID-19 disruptions, which significantly affected health care delivery systems, while constraining malaria control funding, including the distribution of insecticide-treated bed nets (ITNs), indoor-residual spraying (IRS), and treatment [4, 5].

In sub-Saharan Africa (SSA), malaria transmission is mediated by complex interactions between humans, and infected mosquitoes, exacerbated by the favourable physical environments for mosquito survival, and breeding, opportunities for human exposure to mosquito bites, poor healthcare systems, inadequate malaria control interventions [1, 6, 7], as well as land use and land cover changes [8]. Malaria infections can even be more devastating among the structurally disadvantaged populations (i.e. refugees, internally displaced, and asylum-seekers) who live in confined settlements characterized by poor sanitation, poor housing infrastructure, limited access to health care services, inadequate malaria vector control, and economic deprivation [9, 10]. Considering the complexity of malaria transmission dynamics, modelling the determinants of malaria presents numerous challenges in regards to inclusion of uncertainties, non-linearity, and dynamism [11]. It is thus paramount to apply integrated robust models that consider malaria transmission dynamics, to guide pre-emptive policies, and targeted actions for malaria control, and optimal use of resources in the refugee settlements of Uganda, and other refugee hosting countries in Africa.

In most malaria studies conducted in SSA, logistic regression models have been widely used by different scholars to analyse malaria risk factors. For instance, a recent systematic review by Obasohan and colleagues focusing on the period between January 1990 and December 2020 [6], revealed that logistic regression models have been extensively utilized to identify statistically significant malaria risk factors including the nature of housing materials, household wealth status, possession of ITNs, mother’s level of education, environmental resources, drinking water sources and sanitary conditions. In refugee geographical settings, researchers have also used logistic regressions to examine malaria risk factors. For-example, a study conducted in Tongogara refugee camp in Zimbabwe used a logistic regression model, and revealed that housing structures, outdoor activities, and wearing clothes that do not cover the whole body, increased the risk of contracting malaria [12]. Another study conducted in Kiryandongo refugee camp in Uganda also utilized a logistic regression model, and concluded that *Plasmodium falciparum* and intestinal parasitic co-infection was associated with malaria and anaemia [13]. A recent study focusing on all the refugee settlements in Uganda also used a logistic regression model, and revealed that the use of pit latrines, open water sources, lack of ITNs, inadequate knowledge on malaria causes, and prevention, were the key drivers of malaria infections among children under-five [14].

Although these, and recent studies provide valuable insights on malaria risk factors in refugee settlements, they have potential limitations. First, the logistic regression models employed in these studies were used to measure the statistical significance of each determinant of malaria infections with respect to probabilities (*P-value* < 0.01; < 0.05), without any form of importance ranking to inform malaria control efforts in refugee settlements. Second, logistic regression models have been observed to struggle with restrictive expressiveness, and predictive performance, and sometimes multiplicative interpretation of their generated results is difficult [15]. Third, multiple factors influencing the risk for malaria infections do not act in isolation, but rather in an aggregated format [11]. Fourth, logistic regression models were unable to represent conceptual reasoning [16], or complex interactions [15] among the malaria risk factors that were uncertain, stochastic, nonlinear, and multidimensional. Finally, in these studies, the inclusion criteria (*P < 0.20*) that was used to include variables in multivariable logistic regression, left out some key malaria risk determinants.

In response to the limitations of existing research, this study provides an alternative knowledge-based Bayesian belief network (BBN) modelling approach to holistically analyse, predict, and rank the determinants of malaria infections among children under-5 years in the refugee settlements of Uganda. Among others, the BBN is a key integrated modelling approach [17]. Increasingly, BBNs are becoming popular, because of their probabilistic abilities to model uncertainties, and complex environmental domains [18]. A BBN model has several advantages over logistic regression models. BBNs are: (1) highly transparent; (2) flexible in modelling causal relationships; (3) capable of integrating information from various sources (i.e. experimental data, historical data, and expert opinion), and (4) have the potential to explicitly handle uncertainties, and missing data [18, 19]. Because of their versatility, BBNs have been widely used in prediction, data analysis, updating, diagnosis, optimization, deviation detection, and decision-making based on available information [20]. Despite their increasing application in related malaria studies [21–24], BBNs have not been used to study malaria risk factors in refugee settlements of Uganda, and elsewhere.

Thus, a BBN model was developed and utilized data from the 2018–2019 Uganda Malaria Indicator Survey (UMIS), which is the first national wide malaria survey in Uganda to include households, and people in the refugee settlements [25]. Specifically, this study aimed to: (1) develop a novel, and effective knowledge-based BBN model illustrating the conceptual reasoning, and complex causal relationships among the risk factors for malaria infections among children under-five in refugee settlements of Uganda; (2) predict, and rank the risk factors for malaria infections among children under-five in refugee settlements of Uganda. The study’s contribution to the growing body of literature on malaria is twofold. First, this study contributes to the methodological literature on the comprehensive, and holistic assessment of malaria risk factors using BBN technique in refugee settlements. Second, unlike in the previous studies which focused on eliciting statistical significance of the malaria risk factors, this study ranks the risk factors to inform malaria control interventions efforts in refugee settlements. Ranking, and prioritizing malaria risk factors are crucial for allocating resources to targeted malaria control interventions when operating within a context of limited resources.

## Methods

### Study area and justification

**Fig. 1:**
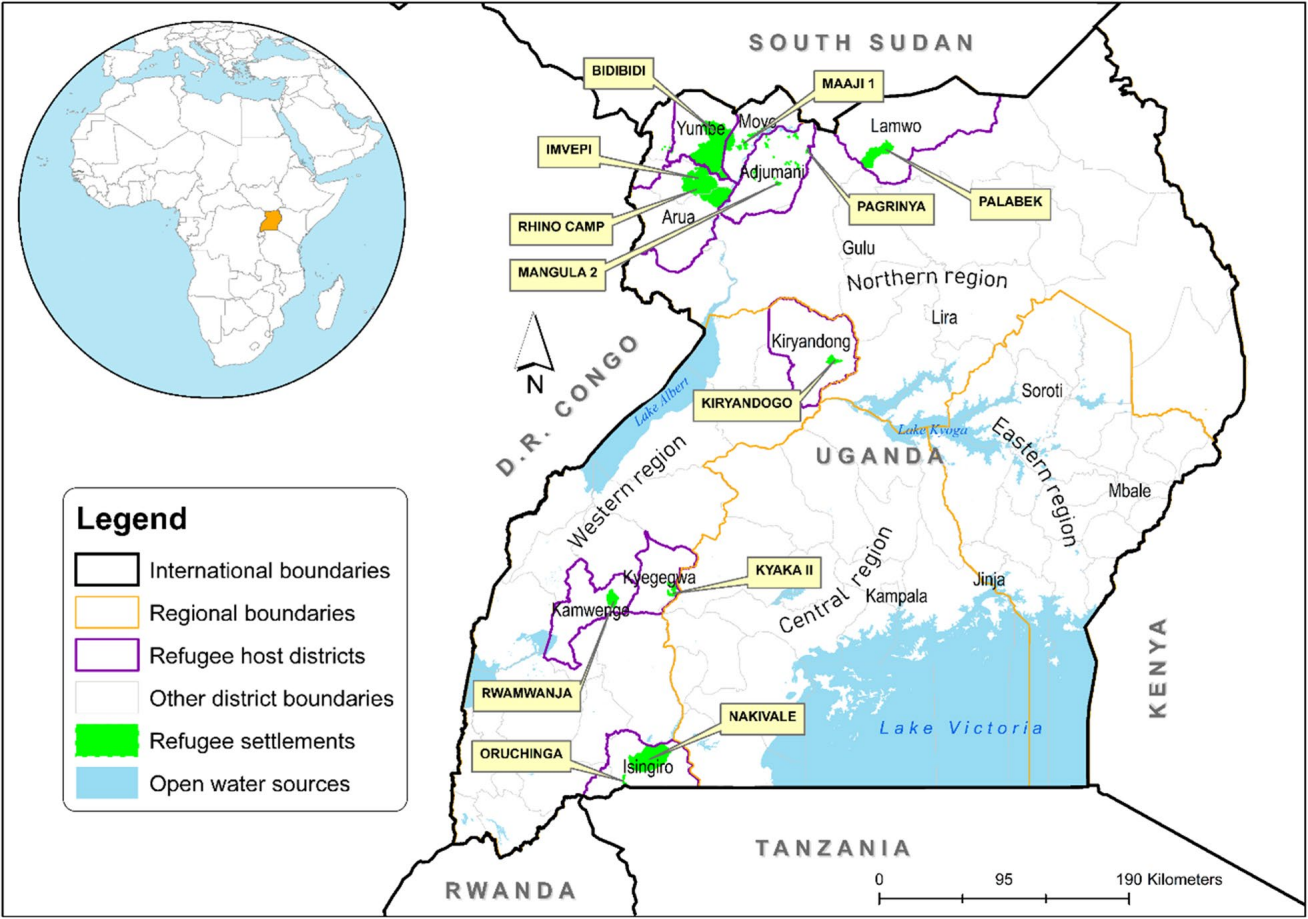
Refugee hosting districts in Uganda

This study focused on refugee settlements located in Uganda (Fig. 1). These settlements are distributed in the districts of Yumbe, Arua, Adjumani, Moyo, Lamwo, Kiryadongo, Kyegegwa, Kamwenge, and Isingiro. Uganda provided an interesting case to comprehensively, and holistically analyse, and rank the risk factors for malaria infections in refugee settlements for several reasons. First, Uganda is the top refugee hosting country in Africa, with over 1.8 refugees coming from mainly Somalia, South Sudan, Democratic Republic of Congo (DRC), and Burundi [26]. Second, Uganda is a malaria endemic country, and by 2021, 5% of 247 million global malaria cases were reported in the country [2]. Third, all refugees in Uganda come from malaria endemic countries, and there is a possibility of Uganda receiving imported malaria strains that might adding an extra burden to the malaria reduction, and elimination efforts [27]. Fourth, Uganda is the only refugee hosting country in Africa collecting malaria related data (i.e., parasite prevalence, anemia, and status of key malaria indicators) via the malaria indicator surveys [25].

### The Bayesian belief network (BBN)

A BBN is a directed acyclic graph (DAG) consisting of a set of variables linked with defined probabilities. A BBN model is widely used for knowledge representation, and reasoning under uncertainty [18, 28]. The DAG represents a qualitative graphical structure, where nodes (i.e., “parent” and “child” nodes) represent the variables of interests that are linked with arrows indicating the existence of probabilistic conditional dependence between two variables. Each node is defined by mutually exclusive states (i.e., categorical, boolean, continuous or discrete), representing alternative choices or conditions for the specific node. The quantitative element of a BBN consists of conditional probability tables (CPTs) corresponding to the nodes having incoming links. The relationships between nodes are described by conditional probability distributions (i.e., priori or unconditional, conditional, and posterior probabilities) that capture the dependences between variables. For-instance, if there is a link going from node A to node C, then A is said to be a “parent node” of C, and C is said to be a “child node” of A. This conditional relationship between the “parent” node A, and “child” node C is defined by a conditional probability table. A BBN model is based on the Bayes’ theorem of probability theory to propagate information between nodes [29]. Bayes’ theorem illustrates how prior knowledge about a given hypothesis X is updated by an observed evidence Y as shown in Eq. 1.

1 $$P(X|Y)=\frac{P(X)*P(Y|X)}{P(Y)}$$

where P(X), is the prior probability of the hypothesis X (i.e., the likelihood that X will be in a particular state, prior to consideration of any evidence), P(Y|X) is the conditional probability (i.e., the likelihood of the evidence, given the hypothesis to be tested); and P(X|Y) is the posterior probability of the hypothesis (i.e., the likelihood that X is in a particular state, conditional on the evidence provided). This equation showing probabilities gives an explicit representation of uncertainties [28].

### Application of the BBN modelling approach to the malaria risk factors

**Fig. 2:**
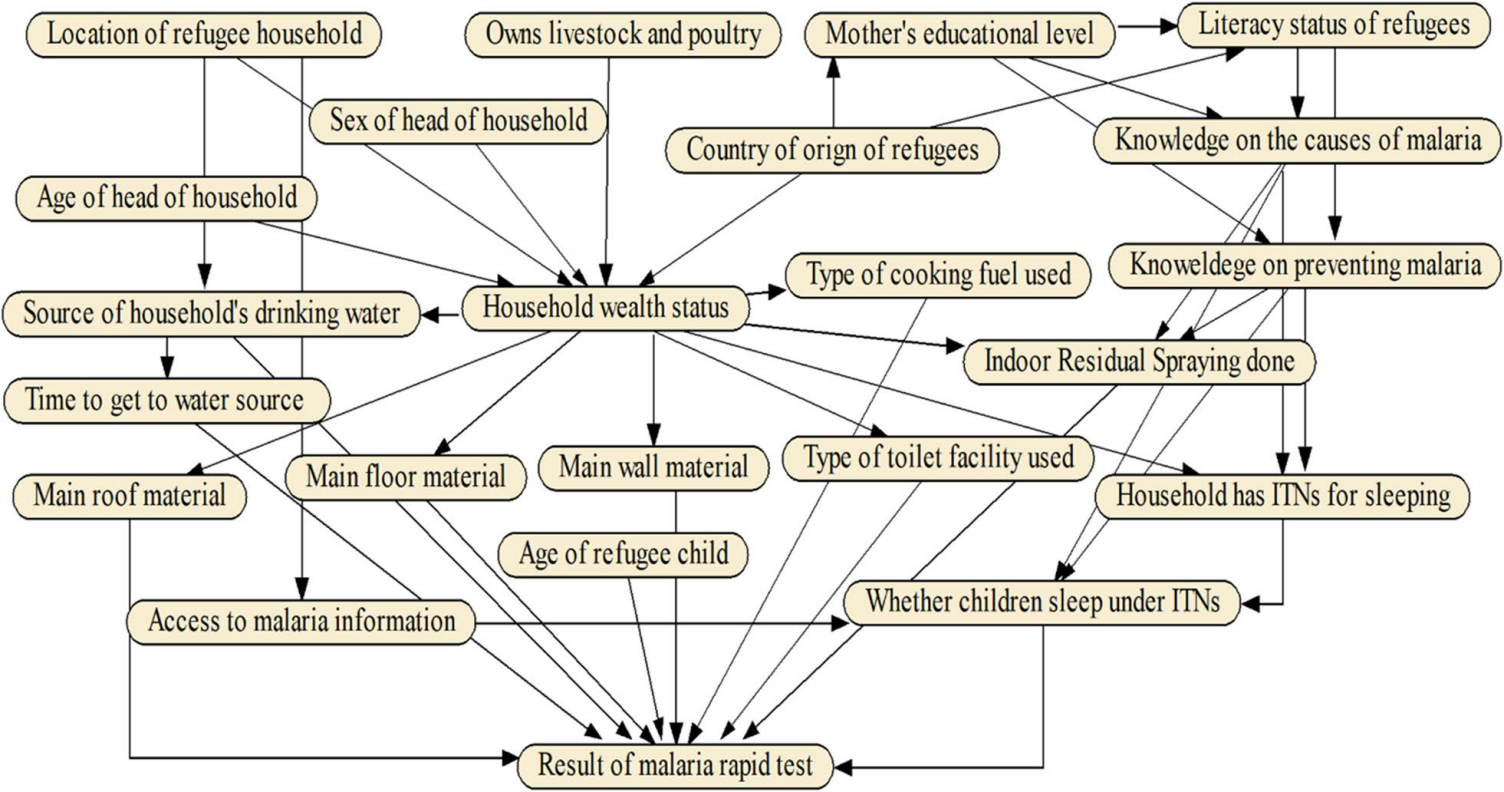
Influence diagram depicting a qualitative causal-effect relationship among the risk factors for malaria infections among children under 5 years in refugee settlements of Uganda

In this study, well established guidelines, and protocols were followed to develop the BBN model [30, 31]. Before constructing a BBN model, it is recommended to either use subject-matter experts or review literature or both to identify key correlates or explanatory variables that influence an outcome of interest [30]. In this study, the outcome of interest was the probability that children under 5 years of age in refugee settlements of Uganda (Fig. 1) tested positive for malaria. To identify the correlates of malaria infections, a literature review was conducted, and the variables deemed relevant for the refugee settlements were identified as shown in Additional file 1: Table S1. Based on literature review, expert knowledge, and the previous BBN modelling experience [21, 22, 32], the risk factors for malaria infections were organized into an influence diagram as shown in Fig. 2.

### Data source, and casefile development for model building

This study utilized a nationally representative data from the 2018–2019 UMIS, and this data was downloaded from the Demographic and Health Surveys programme website [33]. Standardized questionnaires were utilized to collect the demographic, social, economic, and environmental information from the surveyed refugee households. Both rapid diagnostic test (RDT), and the blood smear test (BST) were used to test malaria parasitaemia among children under 5 years with consent obtained from the household heads [25]. The 2018–2019 UMIS involved 3481 children from refugee settlements shown in Fig. 1.

This study focused on 675 children under 5 years who were tested for malaria using the RDT. Microsoft Excel was used to compile a casefile (n = 675) containing the malaria RDT results, and all the variables captured in the influence diagram (Fig. 2). A total of 227 children tested positive for malaria which is equivalent to 33% of the observed malaria prevalence in the full casefile (n = 675).

### Model design, development and parameterization

**Fig. 3:**
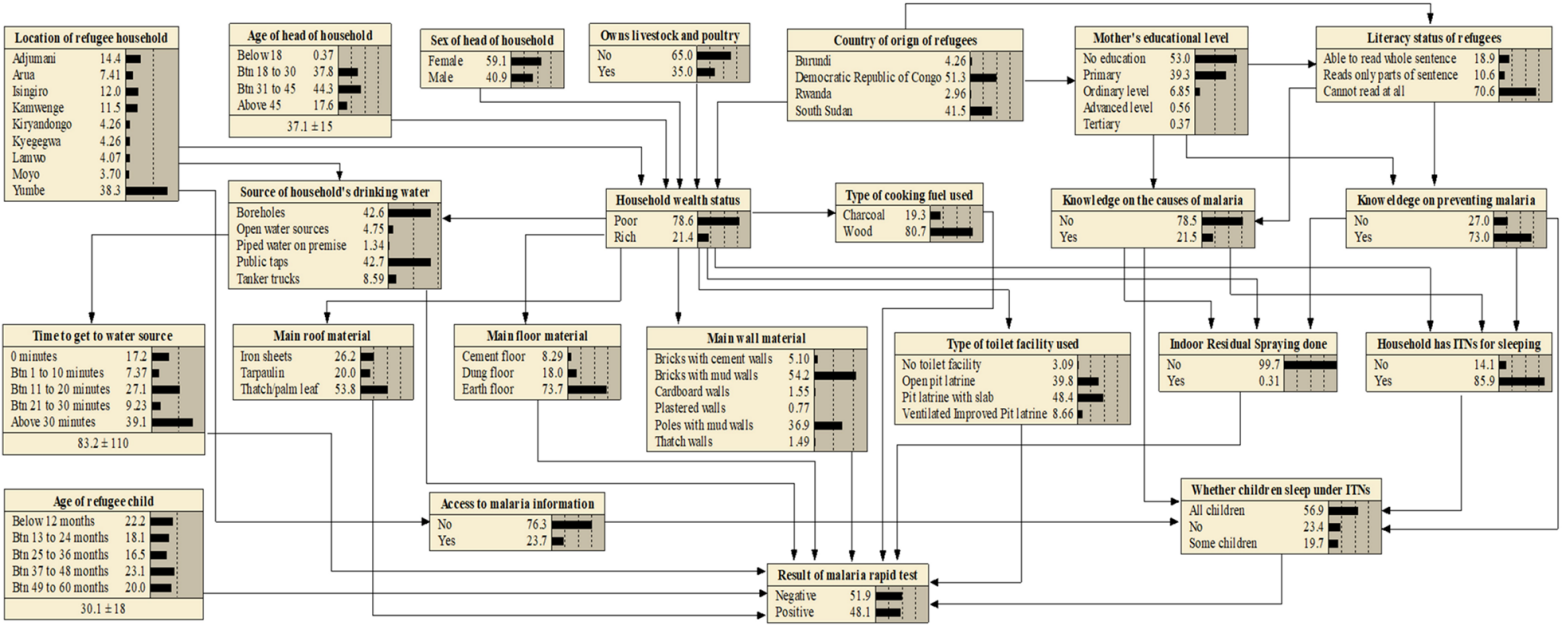
A BBN for modelling the risk factors for malaria infections among children under the age of 5 years in the refugee settlements in Uganda

By using the influence diagram (Fig. 2), a BBN model (Fig. 3) was constructed using Netica software version 6.09 (Norsys Software Corp. Vancouver, Canada). The BBN model structure was determined based on the BBN modelling experience, information from literature (Additional file 1: Table S1), and model reviewers. This combination was adopted to comprehensively, and holistically capture all the malaria risk factors, and to reduce on the model complexity. The model was parameterized based on the variable categories in the questionnaires that were used in the 2018–2019 UMIS.

### Model calibration

Using the K-fold partitioning approach (K = 2), the generated casefile (n = 675) was randomly partitioned into a training portion (80%, n = 540) used to populate the model, and a test dataset (20%, n = 135), which was used to evaluate model performance. A 80/20 data split is among the standard partition ranges recommended for model calibration, and testing [34]. Both the training, and testing datasets were generated using JMP 13 software (JMP Statistical Discovery LLC, North Carolina, USA). The randomly performed K-fold data split generated 182 positive malaria cases for the training dataset (i.e. 33% observed malaria prevalence), and 45 positive malaria cases for the test dataset which also a 33% observed malaria prevalence. The 33% malaria prevalence in both the training, and test datasets is a representative of the 33% observed malaria prevalence in the original dataset (n = 675). The vertical lookup (V-lookup) function in Microsoft Excel was used to extract the randomly partitioned portions (i.e., training, and testing datasets) from the main casefile (n = 675). The developed BBN model was calibrated using the training dataset (80%, n = 540). Learning of the CPTs was based on expectation maximization learning algorithm, a robust technique that automatically updates initial parameter estimates by fitting the data file to the final model [19].

### Model validation

The developed BBN model (Fig. 3) was evaluated using the sensitivity, and prediction performance metrics. Sensitivity analysis can help verify correct initial model structure, and parameterization [29]. It considers that, inputs to the model are uncertain, complex, and provides critical information on how sensitive the performance of the model is to slight or minor changes in the input data [35]. In this study, the function of ‘sensitivity to findings’ in Netica software was invoked to calculate the entropy, and mutual information measures of the BBN model. The entropy measure is based on the assumption that uncertainty or randomness of a variable X, characterized by probability distribution P(X), can be represented by the entropy function as shown in Eq. 2.

2 $$H(X)=-\sum _{i=1}^{n}P(Xi)logP(X)$$

Reducing H(X) by collecting information in addition to the current knowledge about variable X, can be interpreted as reducing the uncertainty about the true state of X. The entropy measure therefore enables an assessment of the additional information required to specify a particular alternative. The mutual information measure was used to assess the impact of obtaining information from variable (Y) in reducing the total uncertainty about variable X using Eq. 3.

3 $$I(Y,X)=H(Y)-H(Y|X)$$

where I(Y, X), is the mutual information between variables. This measure calculates the expected degree to which the joint probability of X, and Y diverges from what it would be if X was independent of Y.

**Fig. 4:**
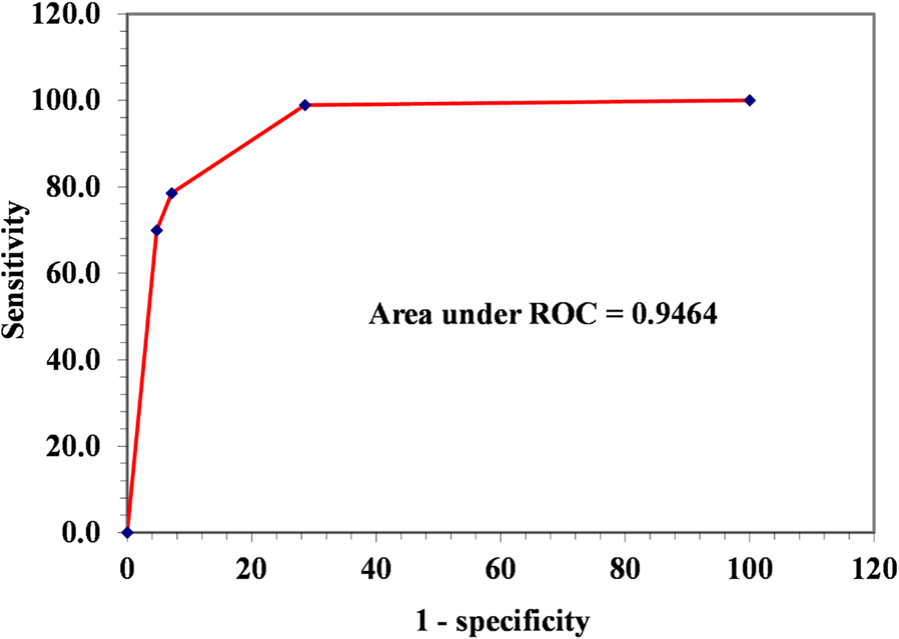
A receiver-operating characteristic curve showing the classification performance of the BBN model

In testing the prediction performance of the BBN model, a ‘test with cases’ function of Netica software was conducted using the generated test dataset (n = 135). In this study, four test metrics were used to evaluate model performance. First, a confusion matrix was used to test the model’s ability to correctly predict both positive, and negative malaria cases among refugee children. Second, to test the classification power of the BBN model, a receiver operator characteristic curve (ROC) was developed in Excel (Fig. 4) based on the sensitivity, and specificity results generated by the model. The ROC was used to assess the model’s prediction accuracy across a continuum of prediction threshold (i.e., 0–100). Besides, the area under the ROC curve (AUC) was also used to measure the overall model performance across a full range of possible cutoffs with value ranges of between 0.5–0.7, 0.7–0.9, and above 0.9 indicating ‘poor’, ‘good’ and ‘excellent’ discrimination abilities respectively. To evaluate the classification success rate of the developed BBN model, the error rate, and scoring rules of logarithmic loss, quadratic loss and spherical payoff were used. For logarithmic loss range (0–infinity), and quadratic loss range (0–2), scores close to zero are considered to be better, whilst 1 indicates the best model performance for spherical payoff (0–1) [29, 35].

## Results

### The Bayesian belief network model

In this study, a BBN model (Fig. 3) was developed to illustrate the conceptual reasoning, and the complex interactions among the risk factors for malaria infections among children under 5 years in refugee settlements in Uganda. The model was compiled using the training dataset (n = 80%) as indicated in Methods section. The BBN model has a total of 23 nodes containing variables (i.e. malaria risk factors) with discrete black state-belief bars indicating the maximum likelihood state. The independent nodes contain prior, and conditional probabilities, and linked together with conditional probability tables. The arrows show the various interactions, direction, and influence of one node to other nodes. The output node (i.e. results of malaria rapid test) show a collective joint effect of all the malaria risk factors expressed as posterior conditional probabilities. The model output results indicate that the probability of refugee children testing positive, and negative for malaria was 48.1%, and 51.9% respectively.

### Model performance

To test the performance of the BBN model (Fig. 3), a total of 135 cases that were randomly selected from the original casefile (n = 675) was used, and the results are shown in Table 1.

**Table 1:** A confusion matrix showing the predication accuracy of the BBN model

	Actual: positive	Scores	Actual: negative	Scores	Total
Predicted: positive	True positive (TP)	38	False positive (FP)	2	40
Predicted: negative	False negative (FN)	4	True negative (TN)	91	95
Total (test dataset)		42		93	135
Model performance	Sensitivity (TP/TP + FN)	0.90	Specificity (TN/TN + FP)	0.98	
	Model error rate	8.89%			
	Logarithmic loss	0.3609			
	Quadratic loss	0.1619			
	Spherical payoff	0.9094			

In Table 1, the confusion matrix shows the number of observed malaria cases that were correctly classified (i.e., True positive malaria cases = 38, and True negative malaria cases = 91). The BBN model has an error rate of 8.89%, implying that, it has an excellent overall accuracy of 91.11% to predict positive, and negative malaria cases correctly. The BBN model’s classification power, was evaluated basing on the scoring rules. The model’s scoring rule results indicated that the model has a strongest predictive power with both the logarithmic loss (0.3609) and quadratic loss (0.1619) scores close to zero, while a spherical payoff (0.9094) approaching 1. The sensitivity results indicated that the BBN model was able to classify 90% of true positive malaria cases correctly. The specificity results further indicate that the model was able to classify true negative cases correctly. To further test the classification ability of the developed model, a receiver-operating characteristic (ROC) curve plotting percentages of true positives against false positives was constructed to assess model accuracy across a range of possible predication cutoffs (Fig. 4).

In Fig. 4, each point on the ROC curve depicts a trade-off between a true positive against a false positive as the cutoff ranges increases from 0.0 to 1.0. The area under the ROC curve which computes the overall performance of the model is 0.9464, implying that a randomly selected child from a malaria positive diagnosis group, had a predicted value larger than that from a child from a malaria negative diagnosis group. The area under the ROC result indicates that the model has an excellent classification ability to distinguish between two diagnostic malaria groups (i.e., positive or negative), much better than a model that randomly classifies malaria cases. With these model performance tests, the developed model is considered to be successful, and appropriate to provide the best interpretation of results, and ranking of the risk factors for malaria infections among children under 5 years in the refugee settlements in Uganda. Extra results on the stability, and generalizability of the developed BBN model in predicting future, and unseen data are shown the Additional file 1: Table S2.

### Ranking of the risk factors for malaria infections in refugee settlements in Uganda

**Fig. 5:**
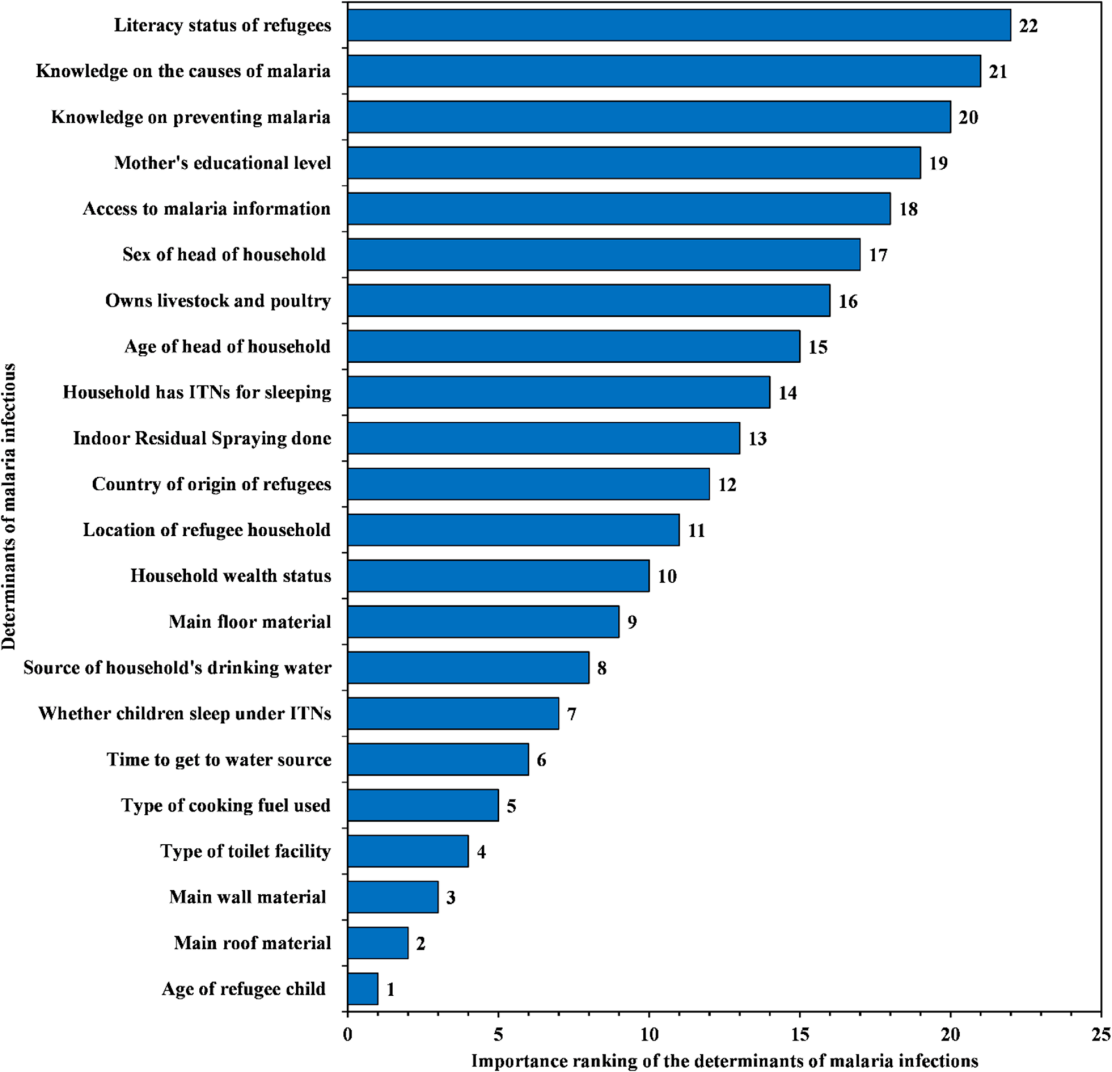
Important ranking of the risk factors of malaria infections in refugee settlements in Uganda

In this study, 22 risk factors for malaria infections (Fig. 3) were considered in the analysis. A sensitivity analysis test was performed on the output node (i.e., results of malaria rapid test) of developed, and tested BBN model (Fig. 3) to rank the relative importance of each risk factor of malaria infection as shown in Fig. 5. Details of the sensitivity analysis results can be found in Additional file 1: Table S3.

In Fig. 5, the first 10 ranked determinants caused the largest entropy reduction in malaria infections in refugee settlements. Although lack ITNs and IRS, age of household head, sex of household head, mother’s level of education, lack of knowledge on the causes, and prevention of malaria have been recently associated with malaria infections in refugee settlements in Uganda [14], in this study, they are not among the 10 ranked determinants because they indirectly influence malaria infections through other factors.

### Prediction of the risk factors of malaria infections in the refugee settlements in Uganda

In this study, the top 10 ranked risk factors (Fig. 5) that contributed to the largest entropy reduction in malaria infections were selected to predict, and estimate their contribution using scenario analysis. In the scenario analysis, the output node of the BBN models (Fig. 3.) was focused on, where the ‘positive’ state belief was tagged to the probability of 100% (i.e. positive malaria diagnosed) to predict the relative changes in the state probabilities of each malaria risk factor as shown in Table 2. Changes in state beliefs were used to calculate percentage point differences for each malaria risk factor to express their contribution to malaria infections among children under 5 years (Table 2). A positive percentage point difference meant that children in refugee settlements were more likely to test positive for malaria. A negative percentage point difference meant that children in refugee settlements were less likely to test positive for malaria.

**Table 2:** Predicted changes in beliefs of states when a positive state finding in the malaria rapid test results node was made 100%

Nodes and states	Ranking	Initial state-beliefs^a^	New state-beliefs^b^	Changes in beliefs^c^	% point change
Malaria test results					
Negative		0.51917			
*Positive*		0.48083	100%		
Age of refugee child	1				
Below 12 months		0.22222	0.21116	− 0.01106	− 1.11
13–24 months		0.18148	0.18231	0.00083	0.08^d^
25–36 months		0.16428	0.17481	0.00947	0.95^d^
37–48 months		0.23148	0.23406	0.00258	0.26^d^
49– 60 months		0.20000	0.20818	0.00818	0.82^d^
Main roof material	2				
Iron sheets		0.26922	0.26216	− 0.00706	− 0.71
Tarpaulin		0.20585	0.19953	− 0.00632	− 0.63
Thatch/palm leaf		0.52493	0.53831	0.01338	1.34^d^
Main wall material	3				
Bricks with cement		0.05297	0.05081	− 0.00216	− 0.22
Bricks with mud		0.54186	0.52887	− 0.01299	− 1.30
Cardboard walls		0.01547	0.01610	0.00063	0.06^d^
Plastered walls		0.00776	0.008043	0.00028	0.03^d^
Poles with mud		0.36904	0.37866	0.00962	0.96^d^
Thatch walls		0.01494	0.01552	0.00058	0.06^d^
Type of toilet facility	4				
No toilet facility		0.03094	0.03216	0.00122	0.12^d^
Open pit latrine		0.39848	0.39005	− 0.00843	− 0.84
Pit latrine with slab		0.48398	0.48743	0.00345	0.34^d^
Ventilated improved pit latrine (VIP)		0.08659	0.09035	0.00376	0.38^d^
Type of cooking fuel	5				
Charcoal		0.19317	0.19992	0.00675	0.67^d^
Fire wood		0.80683	0.80008	− 0.00675	− 0.68
Time to get to water source	6				
0 min		0.17186	0.17270	0.00084	0.08^d^
1–10 min		0.07289	0.07367	0.00078	0.08^d^
11–20 min		0.27082	0.27059	− 0.00023	− 0.02
21–30 min		0.09674	0.09233	− 0.00441	− 0.44
Above 30 min		0.39133	0.38708	− 0.00425	− 0.43
Whether children sleep under ITNs	7				
All children		0.56862	0.56157	− 0.00705	− 0.71
No		0.23421	0.23547	0.00126	0.13^d^
Some children		0.19716	0.20296	0.00580	0.58^d^
Source of drinking water	8				
Boreholes		0.42631	0.42332	− 0.00299	− 0.30
Open water sources		0.04747	0.04865	0.00118	0.12^d^
Piped water on premise		0.01337	0.01375	0.00038	0.04^d^
Public taps		0.42694	0.42462	− 0.00232	− 0.23
Tanker trucks		0.08966	0.08592	− 0.00374	− 0.37
Main floor material	9				
Cement floor		0.08291	0.08584	0.00293	0.29^d^
Dung floor		0.18038	0.18376	0.00338	0.34^d^
Earth floor		0.73671	0.73040	− 0.00631	− 0.63
Household wealth status	10				
Poor		0.78005	0.78593	0.00588	0.59^d^
Rich		0.21995	0.21407	− 0.00588	− 0.59

^a^Represents the initial state belief probabilities for each malaria risk factor in the BBN model

^b^Reflects new state belief probabilities for the malaria risk factors when a positive state belief finding in the output node of the BBN model was made 100%

^c^Indicates the predicted relative changes in probabilities from the initial to new state-beliefs

^d^Represents the actual malaria risk factors, and their positive contribution to malaria infections

From Table 2, refugee households with children aged between 13 and 24 months, 25–36 months, 37–48 months and 49–60 months, had the probabilities of their children testing positive for malaria increasing by 0.08% points (from 18.15 to 18.23%), 0.95% points (from 16.43 to 17.48%), 0.26% points (from 23.15 to 23.41%), and 0.82% points (from 20 to 20.82%), respectively. This finding is consistent with a recent study on malaria risk factors in refugee settlements in Uganda, where it was revealed that children aged between 13 months to 60 months, were more vulnerable to malaria infections compared to children below 12 months [14]. Although the study results indicate that refugee children below 12 months were not vulnerable to malaria, other studies have shown that this age group is equally at a higher risk of malaria infections and should be given extra attention and care [36–39].

In Table 2, refugee households with thatch roof tops had the probability of children testing positive for malaria increasing by 1.34% points (from 52.49 to 53.83%). Roof thatch roof tops are known to be hiding, and resting places for mosquitoes during day time. This finding is consistent with a study conducted on the malaria risk factors in SSA, which revealed that households with thatch roofs had the probability of children testing positive for malaria parasitaemia increasing by 8.61% points [21]. Refugee households with wall constructed with cardboards, plaster, poles and thatch had the probabilities of their children testing positive for malaria increasing by 0.06% points (from 1.55 to 1.61%), 0.03% points (from 0.78 to 0.80%), 0.96% points (from 36.9% to 37/87%), and 0.06% points (from 1.49 to 1.55%) respectively. Households with cement and dung floors, had the probabilities of children testing positive for malaria increasing by 0.29% points (from 8.29 to 8.58%) and 0.34% points (from 18.04 to 18.38%) respectively. This finding is consistent with other studies which have revealed that certain house designs, and building materials used for house construction may increase the malaria risk by enhancing the risk of mosquito entry, density, and survival, indoor mosquito resting, and mosquito bites [40–43].

Refugee households that did not have any toilet facility, had pit latrines with slabs and had VIP latrines, had the probabilities of their children testing positive for malaria increasing by 0.12% points (from 3.09 to 3.22%), 0.34% points (from 48.40 to 48.74%), 0.38% points (from 8.66 to 9.04) (Table 2), respectively. The high malaria risk associated with pit latrines with slabs and VIP latrines is not surprising, since these toilet facilities tend to create conducive resting places and breeding grounds for mosquitoes [44].

From Table 2, refugee households who spent 0 min and between 1 and 10 min walking to the water sources, had the probability of their children testing positive for malaria increasing by 0.08% points (from 17.19 to 17.27%), and 0.08% points (from 7.29 to 7.37%), respectively. Households whose main sources of drinking water were open water sources, and piped water on premises, had the probabilities of their children testing positive for malaria increasing by 0.12% points (from 4.75 to 4.87%), and 0.04% points (from 1.34 to 1.38%) respectively. Open water sources near households and piped water systems, which are poorly managed have been associated with creating potential breeding sites that shortened the gonotrophic cycles while increasing malaria transmission [14, 44]. However, as walk time distance to water sources increases, malaria infections tend to reduce due to prolonged gonotrophic cycles attributed to limited long-range flight abilities of mosquitoes [45]. Refugee households whose children did not sleep under ITNs or had some children sleeping under ITNs, had the probabilities of their children testing positive for malaria increasing by 0.13% points (from 23.42 to 23.55%) and 0.58% points (19.72–20.30%), respectively (Table 2).

## Discussion

Despite extensive research on malaria, the disease remains a major health challenge in many countries of SSA attributed to various socio-economic, and environmental factors. The household level risk factors of malaria infections are known to be complex, stochastic, nonlinear, multidimensional, and do not act in isolation [21]. In refugee settlements, these determinants are also linked to a range of closely related factors including poverty, low levels of education, low access to basic social services, inadequacy of some public policies, racism, sexism, and economic deprivation [9, 46]. Thus, integrated models [17] that consider all these factors are urgently required to enable decision-makers, and stakeholders to draw appropriate conclusions in malaria control interventions in refugee settlements. The recent attempt to model household level determinants of malaria infections in refugee settlements in Uganda [14] was based on a logistic regression model which is not able to fully capture dependencies, uncertainties, complex interactions and ranking of the various malaria risk factors to inform and direct policy interventions on malaria control [11]. The same logistics regression models have been widely used to determine the significant malaria risk factors in many countries of SSA [6, 11, 47].

Here for the first time, a knowledge-based BBN modelling approach has been presented as a potential method to clarify the holistic understanding of the complex interactions among the risk factors of malaria infections among children under 5 years in refugee settlements in Uganda, and quantify the impact of various malaria risk factors. Although a BBN modelling technique has been used to model household factors influencing the risk of malaria among children under 5 years in SSA [21], this is the first study to use a BBN approach focusing on refugee settlements, which are unique given the fact that there are inhabited by structurally disadvantaged populations [46]. Moreover, these structurally disadvantaged populations characterized by racism, sexism, and economic deprivation [46] can lead to further geographical distribution of parasites (i.e. introducing new parasite strains in new locations), cause re-emergence or re-infections as well as lead to multiple, and co-infections with various populations of malaria parasites [14].

Basing on the BBN classification categories (i.e. alpha, beta and gamma) proposed by Marcot et al. [29], the developed BBN model can be considered as a gamma-level model or final application model containing well tested, calibrated, validated, and updated state beliefs with reliable, and accurate probabilistic results which can further be used to inform policy in malaria control programmes in refugee settlements of Uganda. The graphical representation of the model with summarized results in a visually attractive and easy-to-analyse format can be used as part of decision analysis tool in malaria interventions in refugee settlements. The explicit recognition of uncertainty by the developed BBN model can help decision-makers to identify the risks associated with different malaria intervention strategies.

In this study, the risk factors of malaria infections among children under 5 years in refugee settlements in Uganda were ranked in their order of importance (Fig. 5). This is a major advantage of a BBN-modelling structure over traditional statistical models [18, 28]. In Table 2, the predications and estimates provided indicate specific areas which need interventions. The top ranked 10 determinants (i.e., age of child, main roof, wall and floor materials, whether children sleep under ITNs, type of toilet facility used, walk time distance to water sources, type of cooking fuel used, drinking water sources and household wealth) had a higher probability of contributing to malaria burden in refugee settlements. Although lack of ITNs, and IRS, age of household head, sex of household head, mother’s level of education, lack of knowledge on the causes and prevention of malaria have been associated with malaria infections among children under 5 years in SSA as shown in the recent review study [1], in this study, there are not among the 10 ranked determinants in refugee settlements of Uganda. This is because refugee settlements are occupied by structurally disadvantaged populations [46] coming from diverse social-cultural and economic backgrounds which in turn may have varying impact on malaria infections among children.

The top 10 ranked determinants (Fig. 5) are crucial in enhancing the mosquito survival, biting and feeding, parasite development, and breeding [1, 44]. The vulnerability of refugee children to malaria infections is dependent on parents’ personal behaviours, gender roles, physical and environmental factors, social-cultural aspects, and access rights [9]. Ranking and prioritizing risk factors of malaria infections in refugee settlements rather than providing their statistical significance is an important component because, it helps to allocate resources to malaria control interventions within the constraint of limited humanitarian funding [48]. Moreover, ranking and prioritization of malaria risk factors is crucial to provide targeted interventions, since the health services in malaria-endemic countries have had to re-allocate funding and resources towards COVID-19 containment efforts [4].

## Strength and limitations of the study

The study’s main strength is its utilization of a new novel BBN modelling approach that exploited the nationally representative data to generate new evidence, and ranking of the risk factors of malaria infections among children in refugee settlements in Uganda. Thus, model results can be used for targeted malaria control interventions. Despite this strength, this study had some limitations. Although the influence of climate change, environmental factors, land use, and land cover changes on malaria transmission dynamics [8, 22], was recognised, this study did not incorporate these factors, because BBN model was compiled with casefiles generated using non-spatial data extracted from the 2018–2019 UMIS.

## Conclusion

Targeted interventions, and resource allocation are essential for effective malaria control in refugee settlements in Uganda, with predictive integrated models providing important information for decision-making. A BBN model can be used for accurate malaria prediction, and ranking of malaria risk factors. The developed BBN model has an accuracy rate of 91.11% of predicting 48.1% positive, and 51.9% negative malaria cases correctly among children under 5 years in refugee settlements of Uganda. Unlike in the previous studies that focused on the statistical significance of malaria risk factors, the sensitivity analysis results in this study identified, and ranked the malaria risk factors which is an excellent approach to inform policy recommendations on strategic malaria control interventions. The top ranked risk factors of malaria infections included: (1) age of child, (2) roof materials (i.e. thatch roofs), (3) wall materials (i.e., cardboard walls, plastered walls, poles with mud, and thatch wall), (4) whether children slept under ITNs, (5) type of toilet facility used (i.e., no toilet facility, pit latrines with slabs, and VIPs), (6) walk time distance to water sources (i.e., between 0 and 10 min), (7) type of cooking fuel used (i.e., charcoal), (8) drinking water sources (i.e., open water sources, and piped water on premises), and (9) household wealth status (i.e., poor). These results can aid in the identification of priority measures to reduce mosquito density, survival, breeding, mosquito biting rates and human vector contact in refugee settlements. Future studies can focus on the development of a GIS-BBN model that can take into account the Global Positioning System datasets of the 2018–2019 UMIS, and other spatio-temporal environmental, and climate data to disclose interesting features of the malaria transmission hotspots. Risk mapping will captivate the spatial-regional malaria dimension of risk factors in refugee settlements of Uganda within a context of climate change.

### Supplementary Information

**Additional file 1: Table S1.** Household level risk factors associated with malaria infections among children. **Table S2.** A confusion matrix showing the predication accuracy of the BBN model based on the training dataset. **Table S3.** Sensitivity analysis results ranked in decreasing order of influence on model output node based on mutual information and entropy reductions.

## Data Availability

The data used in this study can be obtained by sending a request via the DHS Program website and upon approval data can be obtained from https://dhsprogram.com/data/dataset/Uganda_MIS_2018.cfm?flag=1.

## Abbreviations

AUC: Area under the curve
BBN: Bayesian belief network
BST: Blood smear test
COVID-19: Coronavirus disease 2019
DHS: Demographic and health surveys
GIS: Geographical information system
IRS: Indoor residual spraying
ITNs: Insecticide-treated bed-nets
RDT: Rapid diagnostic test
ROC: Receiver-operating characteristic curve
SSA: Sub-Saharan Africa
UMIS: Uganda Malaria Indicator Survey
VIP: Ventilated improved pit latrines
WHO: World Health Organization
