# Supplementary material for: Bayesian belief network modelling approach for predicting and ranking risk factors for malaria infections among children under 5 years in refugee settlements in Uganda
Source: Malar J. 2023 Oct 4;22:297. doi: 10.1186/s12936-023-04735-8 (PMC10552276; doi:10.1186/s12936-023-04735-8)
Supplement: Supplementary file 1 — Additional file 1: Table S1. Household level risk factors associated with malaria infections among children. Table S2. A confusion matrix showing the predication accuracy of the BBN model based on the training dataset. Table S3. Sensitivity analysis results ranked in decreasing order of influence on model output node based on mutual information and entropy reductions. [file 12936_2023_4735_MOESM1_ESM.docx]

**Bayesian belief network modelling approach for predicting and ranking risk factors for malaria infections among children under five years in refugee settlements in Uganda**

Henry Musoke Semakula^1,2,3,a^**_,_* Song Liang*^3,b^*, Paul Isolo Mukwaya^1,c^, Frank Mugagga^1,d^, Denis Nseka^1e^, Hannington Wasswa^1,f^, Patrick Mwendwa^5,g,^ Patrick Kayima^1,h^, Simon Peter Achuu^4,i^, Jovia Nakato^1,j^

^1^ Department of Geography, Geo-informatics and Climatic Sciences, Makerere University, P.O Box 7062, Kampala, Uganda

^2^Department of Environmental and Global Health, College of Public Health and Health Professions, University of Florida, 2055 Mowry Rd Gainesville FL 32610, USA

^3^Department of Environmental Health Sciences, School of Public Health & Health Sciences, University of Massachusetts, Amherst 01003, USA

^4^National Environmental Management Authority (NEMA), Uganda, Plot 17/19/21 Jinja Road, P.O. Box 22255, Kampala, Uganda

^5^Jomo Kenyatta University of Agriculture and Technology, Department of Horticulture and Food Security. P.O. Box 62000 – 00200 Nairobi.

^a^Tel. (+256) 779-846-203; (+1)352-709 9310; E-mail: [henry.semakula@mak.ac.ug](mailto:henry.semakula@mak.ac.ug), semhm2000@yahoo.co.uk

^b^Tel. (+1) [352 273-9203](mailto:352%20273-9203); E-mail: [songliang@umass.edu](mailto:songliang@umass.edu)

^c^Tel. (+256) 708-470-320; E-mail: [paul.isolomukwaya@mak.ac.ug](mailto:paul.isolomukwaya@mak.ac.ug), pmukwaya@gmail.com

^d^Tel. (+256) 772-968-421; E-mail: [frank.mugagga@mak.ac.ug](mailto:frank.mugagga@mak.ac.ug), fmugagga@gmail.com

^e^Tel. (+256) 782-462-298: E-mail: denisnseka1@gmail.com

^f^Tel. (+256) 782-459-399: E-mail: [wasswahans@gmail.com](mailto:wasswahans@gmail.com)

^g^Tel. (+254) 725 241 797: E-mail: [pmwendwa@jkuat.ac.ke](mailto:pmwendwa@jkuat.ac.ke), [patomwanzia@yahoo.com](mailto:patomwanzia@yahoo.com)

^h^Tel. (+256) 706-037-721: E-mail: [kayimapatrick1789@gmail.com](mailto:kayimapatrick1789@gmail.com)

^i^Tel. (+256) 751 702 025: E-mail: [acusacu@gmail.com](mailto:acusacu@gmail.com), [peter.achuu@nema.go.ug](mailto:peter.achuu@nema.go.ug)

^j^ Tel. (+256) 756 630613, Email: nakatojovia2018@gmail.com

^*^Correspondence author. Tel. (+256) 779-846-203; (+1) 352-709 9310; E-mail: [henry.semakula@mak.ac.ug](mailto:henry.semakula@mak.ac.ug), [semhm2000@yahoo.co.uk](mailto:semhm2000@yahoo.co.uk)

***Review of existing knowledge on malaria risk factors***

A comprehensive survey of relevant literature on malaria risk factors was conducted. The search for literature focused on review papers and scientific research articles. The search was done in four databases namely MEDLINE (Pubmed®), EMBASE®, Scopus®, and Web of Science®. Google scholar and Mendeley search engines were also used to obtain relevant literature. In both the databases, and search engines, keywords, and combination of key words were used to guide the literature search. The search was limited to literature published between 2018 and 2022. Literature was imported into mendeley referencing software and a detailed review was conducted. Explanatory variables identified from the review, and deemed relevant for the refugee settlements are summarized in Table S1.

**Table S1** Household level risk factors associated with malaria infections among children

| **Explanatory variables** | **Description** | **Relationships with malaria infections** | **Sources** |
| --- | --- | --- | --- |
| **S**ex of household head | Determines the ability to obtain malaria treatment and prevention measures | **+** | [1–3] |
| Mother's education/Literacy levels | Determines acquisition and usage of malaria prevention methods and treatment | **+** | [1, 3–5] |
| Location of households | Provides adequate source of blood meal for mosquitos. | **+** | [1, 3, 4] |
| Household wealth status | Influences housing quality, ownership and payment for malaria prevention and treatment | **+** | [1, 3–5] |
| Insecticide treated nets (ITNs) | Prevents night mosquito bites around the beds | **+** | [1–4, 6, 7] |
| Indoor residual spraying (IRS) | Kills or repels mosquitoes which feed and rest indoors | **+** | [1–4, 6, 7] |
| Wall materials | Influences the level at which mosquitoes enter households | **+** | [1, 3–5, 7–9] |
| Roof materials | Determines the suitability of indoor resting sites for mosquitoes | **+** | [1–7] |
| Floor materials | Influences indoor mosquito density | **+** | [1–7] |
| Sanitation | Determines the nature of breeding sites for mosquitoes around households | **+** | [1, 3, 4, 8] |
| Drinking water sources | Influences the nature of ovi-position sites and the time required by vectors to locate them. | **+** | [1, 3, 4, 8] |
| Distance to water sources | Determines the time taken by mosquitoes to find a suitable breeding site. | **+** | [1, 3, 8] |
| Type of cooking fuel | Influences indoor mosquito density, survival and biting rates | **+** | [3, 8] |
| Knowledge of the cause and prevention of malaria | Influences the likelihood of ITN use and malaria treatment | **+** | [10] |
| Livestock owned | May retard or divert mosquitoes from biting people | **+** | [8–10] |
| Access to malaria information | Influences the likelihood of ITN use and malaria treatment | **+** | [8] |

**Table S3.** A confusion matrix showing the predication accuracy of the BBN model based on the training dataset

|  | **Actual: Positive** | **Scores** | **Actual: Negative** | **Scores** | **Total** |
| --- | --- | --- | --- | --- | --- |
| Predicted: Positive | True Positive (TP) | 42 | False Positive (FP) | 8 | 50 |
| Predicted: Negative | False Negative (FN) | 140 | True Negative (TN) | 350 | 490 |
| Total (Test dataset) |  | 182 |  | 358 | 540 |
| Model Performance | Sensitivity (TP/TP+FN) | 0.23 | Specificity (TN/TN+FP) | 0.98 |  |
|  | Model Error rate | 13.79% |  |  |  |
|  | Logarithmic loss | 0.9788 |  |  |  |
|  | Quadratic loss | 0.435 |  |  |  |
|  | Spherical payoff | 0.7527 |  |  |  |

**Sensitivity results**

**Table S3**. Sensitivity analysis results ranked in decreasing order of influence on model output node based on mutual information and entropy reductions

| **Determinants** | **Mutual information** | **Percent reduction** | **Variance of beliefs** |
| --- | --- | --- | --- |
| Age of refugee child | 0.00062 | 6.2 | 0.000213 |
| Main roof material | 0.00048 | 4.8 | 0.000167 |
| Main wall material | 0.00046 | 4.6 | 0.000159 |
| Type of toilet facility | 0.00028 | 2.8 | 0.000096 |
| Type of cooking fuel used | 0.00020 | 2.0 | 0.000068 |
| Time to get to water source | 0.00018 | 1.8 | 0.000062 |
| Whether children sleep under ITNs | 0.00018 | 1.8 | 0.000061 |
| Source of household's drinking water | 0.00016 | 1.6 | 0.000055 |
| Main floor material | 0.00015 | 1.5 | 0.000051 |
| Household wealth status | 0.00014 | 1.4 | 0.000048 |
| Location of refugee household | 0.00003 | 0.3 | 0.000009 |
| Country of origin of refugees | 0.00001 | 0.1 | 0.000002 |
| Indoor Residual Spraying done | 0.00000 | 0.0 | 0.000001 |
| Household has ITNs for sleeping | 0.00000 | 0.0 | 0.000001 |
| Age of head of household | 0.00000 | 0.0 | 0.000000 |
| Owns livestock and poultry | 0.00000 | 0.0 | 0.000000 |
| Sex of head of household | 0.00000 | 0.0 | 0.000000 |
| Access to malaria information | 0.00000 | 0.0 | 0.000000 |
| Mother's educational level | 0.00000 | 0.0 | 0.000000 |
| Knowledge on preventing malaria | 0.00000 | 0.0 | 0.000000 |
| Knowledge on the causes of malaria | 0.00000 | 0.0 | 0.000000 |
| Literacy status of refugees | 0.00000 | 0.0 | 0.000000 |

**References**

[1] Boyce MR, Katz R, Standley CJ. Risk factors for infectious diseases in urban environments of sub-Saharan Africa: A systematic review and critical appraisal of evidence. Trop Med Infect Dis 2019;4..

[2] Ahmed S, Reithinger R, Kaptoge SK, Ngondi JM. Travel is a key risk factor for malaria transmission in pre-elimination settings in Sub-Saharan Africa: A review of the literature and meta-analysis. Am J Trop Med Hyg 2020;103:1380–7..

[3] Obasohan PE, Walters SJ, Jacques R, Khatab K. A scoping review of the risk factors associated with anaemia among children under five years in sub-Saharan African countries. Int J Environ Res Public Health 2020;17:1–20..

[4] Degarege A, Fennie K, Degarege D, Chennupati S, Madhivanan P. Improving socioeconomic status may reduce the burden of malaria in sub Saharan Africa: A systematic review and meta-analysis. PLoS One 2019;14:1–26..

[5] Vilcins D, Sly PD, Jagals P. Environmental Risk Factors Associated with Child Stunting: A Systematic Review of the Literature. Ann Glob Heal 2018;84:551–62.

[6] Furnival-Adams J, Olanga EA, Napier M, Garner P. Housing interventions for preventing malaria. Cochrane Database Syst Rev 2019;2019..

[7] Furnival-Adams J, Olanga EA, Napier M, Garner P. House modifications for preventing malaria. Cochrane Database Syst Rev 2021;2021..

[8] Semakula HM, Liang S, Mukwaya PI, Mugagga F, Swahn M, Nseka D, et al. Determinants of malaria infections among children in refugee settlements in Uganda. Analysis of data from Uganda malaria indicator survey, 2018-19. Infect Dis Poverty 2023;3:1–12.

[9] Semakula HM, Song G, Achuu SP, Zhang S. A Bayesian belief network modelling of household factors influencing the risk of malaria: A study of parasitaemia in children under five years of age in sub-Saharan Africa. Environ Model Softw 2016;75:59–67.

[10] Semakula HM, Song G, Zhang S, Achuu SP. Potential of household environmental resources and practices in eliminating residual malaria transmission: A case study of tanzania, burundi, malawi and liberia. Afr Health Sci 2015;15..
